# Protein functional site annotation using local structure embeddings

**DOI:** 10.1073/pnas.2513219122

**Published:** 2025-08-20

**Authors:** Alexander Derry, Alp Tartici, Russ B. Altman

**Affiliations:** ^a^Department of Biomedical Data Science, Stanford University, Stanford, CA 94305; ^b^Department of Genetics, Stanford University, Stanford, CA 94305; ^c^Departments of Medicine, Stanford University, Stanford, CA 94305

**Keywords:** protein function, functional site, machine learning, explainability

## Abstract

The rapid expansion of protein sequence and structure databases has resulted in a significant number of proteins with ambiguous or unknown function. While advances in machine learning techniques hold great potential to fill this annotation gap, current methods for function prediction are unable to associate global function reliably to the specific residues responsible for that function. We address this issue by introducing PARSE (Protein Annotation by Residue-Specific Enrichment), a knowledge-based method which combines pretrained embeddings of local structural environments with traditional statistical techniques to simultaneously predict function and provide residue-level annotations. For the task of predicting the catalytic function of enzymes, PARSE achieves comparable or superior global performance to state-of-the-art machine learning methods (F1 score > 85%) while simultaneously annotating the specific residues involved in each function with much greater precision. Since it does not require supervised training, our method can make one-shot predictions for very rare functions and is not limited to a particular type of functional label (e.g. Enzyme Commission numbers or Gene Ontology codes). Finally, we leverage the AlphaFold Structure Database to perform functional annotation at a proteome scale. By applying PARSE to the dark proteome—predicted structures which cannot be classified into known structural families—we predict several bacterial metalloproteases. Each of these proteins shares a strongly conserved catalytic site despite highly divergent sequences and global folds, illustrating the value of local structure representations for new function discovery.

## Significance

Computational methods for protein function annotation are necessary in order to keep up with the quantity of new sequences and structures being deposited in protein databases. Despite the good predictive performance of machine learning-based predictors, their practical utility is limited by their inability to identify the specific amino acids responsible for each function. Here, we propose an approach which combines the expressivity of deep learning representations of protein structure with the interpretability of knowledge-based statistical methods. This method predicts both overall function and the corresponding catalytic site for enzymes with high precision and residue-level resolution, even for very rare enzyme classes. By annotating unclassified structures in the AlphaFold database, we identify several putative bacterial metalloproteases.

Proteins are complex molecules that perform a diverse range of biochemical functions, including molecular binding and transport, cellular signaling, and reaction catalysis. Identifying the set of functions performed by a protein is critical for elucidating its role in biological processes, which in turn enables greater understanding of disease pathogenesis and more precise targeting of therapeutics. Large-scale sequencing efforts and improvement in both experimental and computational techniques have resulted in the rapid expansion of sequence databases such as the UniProt Knowledgebase (UniProtKB) (1), which has more than doubled in size in the last five years to over 250 million protein sequences. UniProtKB is the primary repository for function annotations, including membership in protein family databases [e.g. Pfam (2), InterPro (3) and classification to controlled terms from ontologies such as the Gene Ontology (GO) (4) or Enzyme Commission (EC) (5)]. However, experimental characterization or expert assessment of a protein’s function is infeasible at such scale, resulting in a significant annotation gap—the manually curated subset of UniProtKB [SwissProt (6) contains less than 0.3% of the full database, and this proportion is rapidly shrinking].

In addition to global assignment of protein function, the identification of amino acids involved in each biochemical action is crucial for understanding a protein’s mechanism of action and to guide protein engineering and design efforts, which are often precisely targeted at specific functional sites. However, here the annotation disparity is even more stark: Over 60% of proteins assigned an enzymatic function (i.e. EC number) in SwissProt have no active site residues identified. Curated databases of residue-level annotations are inherently limited in scope by the effort required to update and maintain them. For example, the Catalytic Site Atlas (CSA) (7), which contains detailed information about the residues involved in the enzyme catalytic mechanisms, is limited to one reference sequence and structure for each curated enzymatic function and is not being regularly updated.

The development of computational methods for predicting protein function is therefore a major challenge in protein science. Domain-specific profile hidden Markov models built on multiple alignments of homologous sequences (8–11) have traditionally been a dominant approach and form the basis of most protein family databases (2, 3, 12). To address the limitations of annotation transfer via homology, machine learning (ML) methods that integrate features from sequence, structure, and/or protein interaction networks have been developed for de novo function classification (13–19). Recent methods have leveraged self-supervised deep learning techniques such as protein language modeling (20–22), which can learn complex patterns from massive datasets without explicit feature engineering, to establish a new state of the art for protein function classification (23–27). However, while ML methods continue to improve, they have several limitations as general-purpose tools for function annotation.

First, to assemble sufficiently large labeled training datasets, many methods rely on predefined labels which are often broad or ambiguous. For example, GO terms have varying levels of granularity and have been shown to be biased toward less-informative annotations from a small number of high-throughput experiments (28, 29). Similarly, although EC numbers are arranged in a four-level hierarchy with a more consistent level of specificity at the lowest level, some EC numbers are so rare that they are either excluded from training or aggregated up to a higher level of the tree. The imbalance in class sizes also results in decreased performance for rare function classes, further exacerbating the bias toward well-studied proteins (30). A recent method, CLEAN (31), improved performance on rare proteins by introducing a contrastive learning procedure. However, updating any supervised model with additional data or new labels requires retraining from scratch, adding overhead and potentially changing its performance characteristics.

Second, as sequence databases expand to species from across the tree of life (e.g. microbial metagenomes), it is important to be able to accurately annotate sequences that have low similarity to previously studied proteins. Methods which operate directly on protein structure provide a natural solution to this issue since structure is much more conserved than sequence and the biochemical activities of a protein in the cell are determined directly by its 3D conformation. However, the utility of such methods for function annotation has been limited by the availability of high-quality structure data, both in the context of training models (limited structures with functional labels) and of applying them at scale (most proteins of unknown function have only sequence available). Recent advances in structural biology have greatly increased the number of experimental structures in the Protein Data Bank (PDB) (32), allowing for large-scale function prediction models to be trained directly on 3D structure. For example, DeepFRI (24) combines a spatial graph of residues in the structure with sequence-based features from a protein language model. Additionally, the release of high-quality predicted structures for hundreds of millions of proteins (22, 33, 34) provides the opportunity to apply structure-based function prediction models to unannotated proteins at an unprecedented scale. These methods have already resulted in the discovery of novel structural folds in need of annotation (35–37).

Third, methods for global prediction neglect the problem of residue-level annotation. As a result, local annotations are typically made using separate models built specifically for each functional site. These may be based on sequence motifs (38), manually defined rules (39, 40), or local structural representations (41–43), but they are all inherently limited by the need to individually develop a model for each function as well as a method for scanning over a protein of interest to discover potential hits (44). Some global ML methods, including DeepFRI and ProteInfer (27), attempt to identify key amino acids using class activation mapping (45), which uses the gradients of the trained model post hoc to identify regions of the input which contribute to the prediction. However, these explainability techniques tend to be imprecise at a residue level, are not robust to spurious correlations (46, 47), and are typically evaluated qualitatively. Moreover, without reliable identification of functional residues the global predictions themselves may be misleading; for example, consider an enzymatic domain which is lacking a single catalytic residue critical to its function and is therefore inactive. Indeed, the lack of methods which can make accurate global predictions and provide residue-level explainability is cited as a major reason why few newly developed functional predictors are widely adopted by experimental biologists (28). Concretely, in this work, we consider explainability to refer to the ability of a model to justify its protein-level function predictions by identifying the residues which are associated with that function in a human-comprehensible manner.

In light of these limitations, we present PARSE (Protein Annotation by Residue-Specific Enrichment), a knowledge-based method for automated function prediction which 1) predicts specific biochemical function and does not require large amounts of training data, with only one reference example required for each class; 2) leverages rich local structure representations pretrained on evolutionary relationships across the PDB to capture functionally relevant features; and 3) simultaneously provides both a global function prediction and the individual residues which contribute to the prediction (48). Focusing on the task of enzymatic function prediction, we show that PARSE achieves comparable performance on global classification to current methods while simultaneously providing high-precision annotations of residues in the catalytic site. Using the AlphaFold Structure Database (AlphaFoldDB), we expand annotation coverage in the human proteome and provide functional hypotheses for several structural folds in the “dark proteome,” structures which do not share significant similarity to any annotated proteins. PARSE is open-source and available as a command-line Python tool at: https://github.com/awfderry/PARSE.

## Results

### PARSE Simultaneously Predicts Global Function and Annotates Key Functional Residues.

**Fig. 1.**
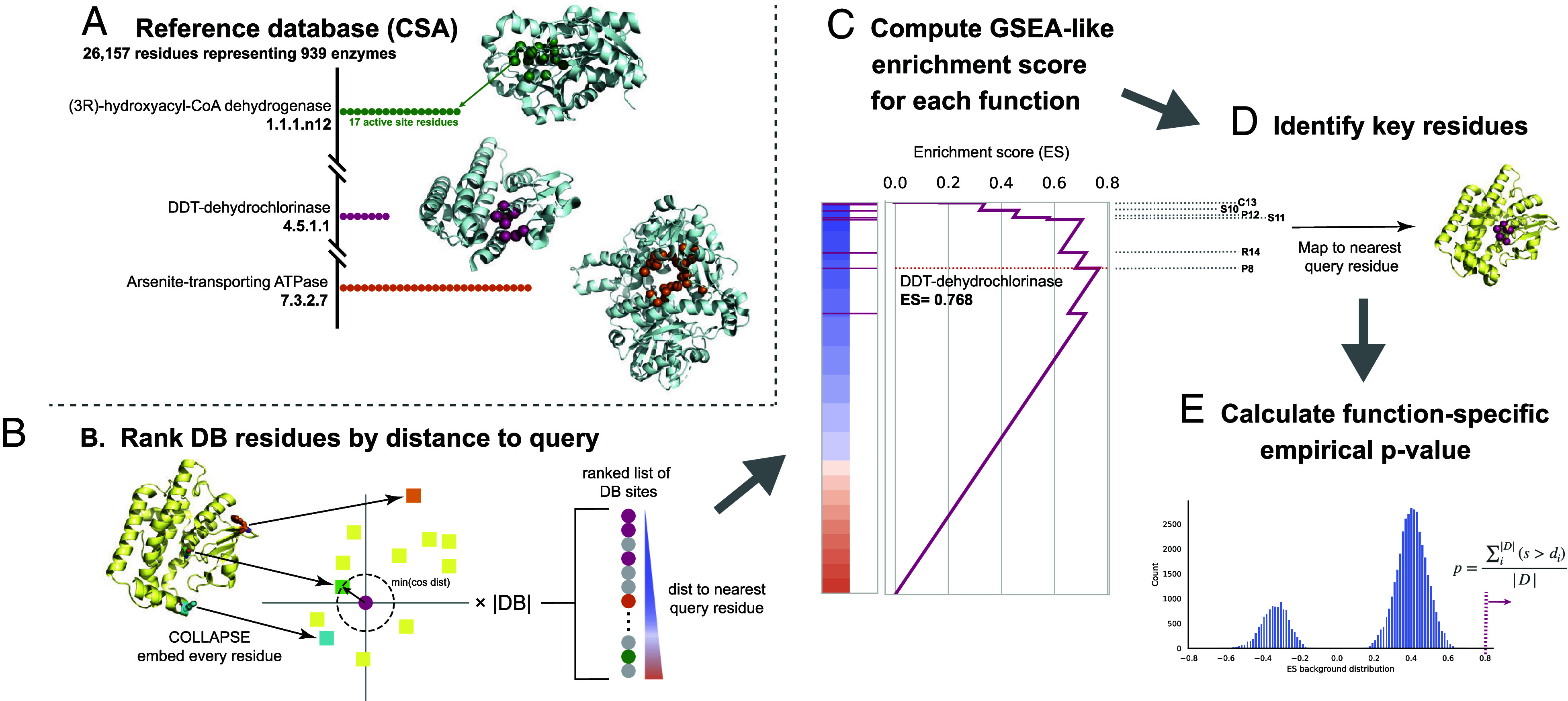
The PARSE algorithm for explainable protein function annotation. Starting from *Top Left*, we first (*A*) build a reference database containing all residues associated with each functional group (here, enzymes from the Catalytic Site Atlas). Then, for a query protein to be annotated, we (*B*) embed the local environment around each residue using COLLAPSE (colored squares) and compute the pairwise cosine distance to the embedding of each residue in the reference database (colored circles). Database residues are then ranked by the minimum distance to any residue in the query and (*C*) an enrichment score is computed for each functional group relative to this ranked list. (*D*) Key residues for a given function are mapped to the query protein using the leading-edge subset of database residues which achieve scores greater than the maximum running enrichment score in the ranked list. Finally, to assess significance and reduce the influence of low-specificity functional labels, we (*E*) compute an empirical *P*-value based on a function-specific background score distribution.

A protein’s function depends largely on the presence of and interactions between several key functional residues and their surrounding structural microenvironments. We take advantage of this to develop PARSE, a knowledge-based method which uses site-level comparisons in a learned embedding space of local protein structure in order to find residues with high similarity to a database of known functional sites. These residue-level similarities are then aggregated using a simple statistical method to identify functions that are enriched at the protein level. Importantly, unlike methods which are based on supervised machine learning techniques, PARSE is capable of identifying arbitrary functional sites even with only one known example and is explainable by construction, supporting every global annotation with the key functional residues which contribute to the prediction being made. Specifically, the PARSE algorithm consists of the following key components (Fig. 1; *PARSE Implementation Details*).

First, we define a database of protein structures, each annotated with both a global functional class and the set of residues which contribute to that function. In this work, we use the CSA (7), a curated database of experimentally validated enzymes with high-quality structures and residue-level data on catalytic activity. We then extract the local structural environment around every functional residue in this reference database using the corresponding crystal structure from the PDB, producing a large set of sites and their corresponding functions (Fig. 1*A*; see *Materials and Methods* for details).

To annotate a query protein using this database, we first compute the pairwise similarities between each site in the database and the local structural environments around each residue in the query. We then rank all reference database residues by their maximum similarity to any query residue (Fig. 1*B*). To efficiently compare local structural environments, we use low-dimensional representations generated by COLLAPSE (43), a deep learning method for embedding local structural sites into a numerical vector space. COLLAPSE embeddings were pretrained in a self-supervised manner using comparisons between evolutionarily related sites across the PDB, enabling them to capture conserved structural and functional features. These embeddings are ideally suited for this task, and we have previously demonstrated that similarity in the embedding space can be used to precisely distinguish between functional sites (43).

Intuitively, if a query protein performs a certain enzymatic function, the catalytic residues corresponding to that function should appear near the top of the ranked list of database sites. Detecting enriched functions in this list is analogous to the problem of Gene Set Enrichment Analysis (GSEA) (49), a widely used method for identifying enriched biological processes in gene expression datasets. Like GSEA, we compute an enrichment score for each function by computing a modified Kolmogorov–Smirnov (K-S) statistic (Fig. 1*C*). The contributing residues for each prediction are identified by mapping the leading-edge subset of database residues back to their nearest correspondences in the query (Fig. 1*D*), and statistically significant predictions are identified using a function-specific empirical *P*-value computed over a validation set derived from SwissProt (Fig. 1*E*). This algorithm is computationally efficient and can annotate a standard 200-residue protein in 15 to 20 s with no specialized hardware (1 CPU with 4 cores) and less than 10 s on a single GPU (compared to 6 to 8 s for DeepFRI and 12 to 15 s for CLEAN).

### Accurate Function Prediction for Known Enzymes.

First, we measured the performance of PARSE for predicting global (protein-level) function relative to best-in-class machine learning predictors. We note that our goal with this evaluation is not to establish a state-of-the-art for global function prediction but to ensure that we achieve competitive performance on protein-level predictions, even for rare enzyme classes, while achieving best-in-class explainability via residue-level annotations. Therefore, we select two baseline methods against which to compare the results of PARSE: CLEAN (state-of-the-art for global function prediction, but no ability to produce residue-level predictions) (31) and DeepFRI (structure-based model with residue-level saliency mapping) (24). For both baselines, we run the models in inference mode using the provided weights out-of-the box. For a simple homology-based baseline, we compare to BLASTp (50). To showcase the merits of the PARSE methodology in comparison to simpler statistical analyses of the COLLAPSE embedding similarities alone, we evaluate the predictive performances of four different baselines that make more direct use of the underlying COLLAPSE embeddings (*Materials and Methods*).

**Fig. 2.**
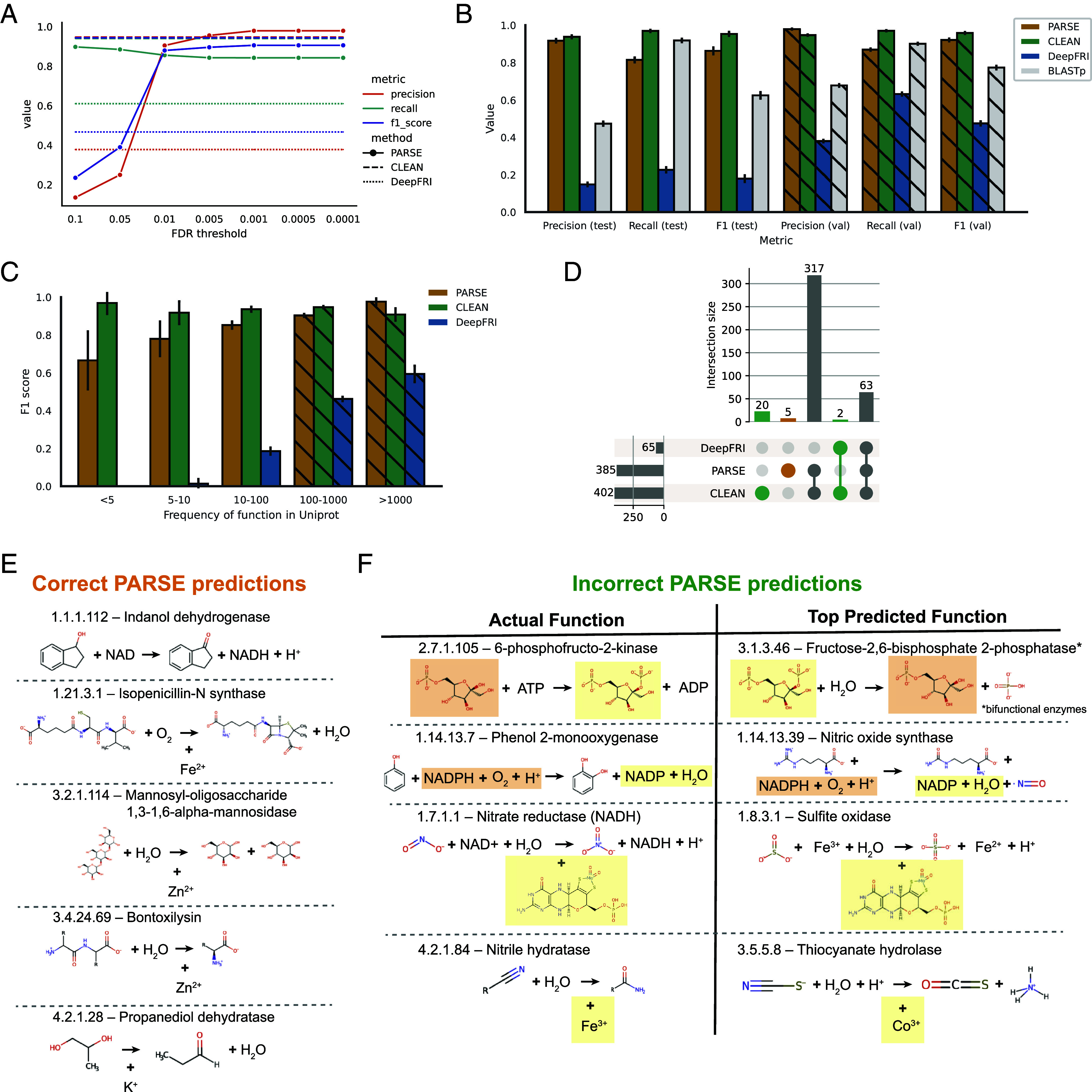
Global prediction performance on enzymes with known function. (*A*) Tuning of FDR-corrected *P*-value threshold on validation set. Performance in terms of precision, recall, and F1 score at each threshold are compared to PARSE and DeepFRI. (*B*) Precision, recall, and F1 score for each method on held-out test set of rare enzyme classes. Validation set performance is shown in the hatched bars for comparison. Error bars represent 95% CIs computed using the Wilson score interval (51). (*C*) F1-score for each method binned by the count of the enzyme class (EC number) in SwissProt. Error bars represent 95% CIs. Enzyme classes with more than 100 examples are in the validation set, shown again using hatched bars. (*D*) Analysis of which enzyme classes are able to be predicted by each method. On the *Left*, the upset plot shows all intersections between the unique EC numbers predicted correctly for each method. The marginal size of each set is shown by the histograms on each axis. (*E*) EC numbers predicted correctly by PARSE only, including the products, reactants, and cofactors involved in the reaction [orange in (*E*)]. (*F*) Error analysis for four sampled functions which were predicted correctly by CLEAN but not PARSE [green in (*E*)]. Products, reactants, and cofactors which are shared between ground truth and prediction are highlighted in yellow and orange.

We evaluated performance using a dataset of 17,262 known enzymes derived from a nonredundant subset of SwissProt, with corresponding structures predicted by AlphaFold2 (33, 34) (*Materials and Methods*). Importantly, most or all of the proteins in our evaluation dataset have already been seen by both DeepFRI and CLEAN in training, inflating their true out-of-distribution performance relative to PARSE. To mitigate this, we selected 3623 proteins with EC numbers represented less than 100 times in SwissProt as a held-out test set for evaluation, expecting that these represent the most challenging cases for supervised models. The remaining 13,639 proteins were used as a validation set to compute empirical score distributions and identify optimal significance thresholds for predicting enzymatic function (Fig. 2*A*). We find that validation performance in terms of protein-level precision, recall, and F1 plateaus at an FDR-corrected *P*-value of 0.001, which we choose as a threshold for all future experiments. At this threshold, PARSE performs similarly to CLEAN, with slightly better precision but lower recall, and significantly outperforms DeepFRI in both metrics.

On the test set, we see similar results, with minimal decrease in performance for both PARSE and CLEAN relative to the validation set (Fig. 2*B*). We also achieve much greater precision than a simple BLASTp search on both validation and test sets, even while achieving comparable recall. PARSE significantly outperforms the simpler COLLAPSE-based baselines in both precision and recall, highlighting the performance gains achieved by the statistical workflow (*SI Appendix*, Fig S7). To assess the performance on rare enzymes specifically, we divided our dataset into five bins based on the number of times that enzyme appears in SwissProt and computed the F1-score for each bin. Even for very rare enzymes with less than five examples in SwissProt, we achieve good performance (F1 = 0.67), while DeepFRI performance drops to zero (Fig. 2*C*). CLEAN achieves consistently high performance due to its contrastive learning objective, even with the caveat that even rare enzymes were seen during training. The increased generalizability to unseen and rare test examples is a key strength of PARSE, reflecting its zero-shot capability and simplicity as a statistical enrichment model relative to complex deep learning models with millions of trainable parameters.

Next, we examined the errors made by each method to better understand their performance characteristics. For proteins that were not annotated correctly by the top-ranked prediction, we quantified whether the method was predicting a similar function or lower level of specificity (i.e. shared third level or higher of the EC hierarchy), whether it was predicting an entirely different function, or whether it made no predictions at all (*SI Appendix*, Fig. S1). We find that the majority of incorrect predictions made by all three methods do indeed share at least one EC number, and DeepFRI is particularly notable for its lack of specificity, with the majority of predictions correct at the third level but not at the fourth (in many of these cases, the full EC number is present but lower-ranked in the list). Additionally PARSE exhibits high precision; while it declines to make a prediction (i.e. no functions achieve statistical significance) in more cases than both DeepFRI and CLEAN, when it does make a prediction, it is more likely to be correct to the fourth EC level.

We also analyzed which individual functions could be predicted by each method (Fig. 2*D*). PARSE and CLEAN show high agreement (77.9% of EC numbers) and all three methods agree on a further 15.5%. There were five functions which only PARSE could identify; notably these seem to be enriched for the presence of metal ions as cofactors, which appear in four of these enzymes (Fig. 2*E*). There were also 22 functions which PARSE could not annotate correctly (Fig. 2*F*). Among these misannotations are a group of bifunctional enzymes where only one function is recognized (fructose-6-phosphate-2-kinase-EC 2.7.1.105/fructose-2,6-biphosphatase-EC 3.1.3.46) (Fig. 2*F*). The remainder of predictions shared either reactants (e.g. ATP, NADPH), products (e.g. ADP, NADP), or cofactors (e.g. metal ions, molybdopterins) with the true catalyzed reaction. This reflects the ability of PARSE to detect functional site similarities via local structure comparisons, even when the precise biochemical reaction may be more difficult to predict. For proteins that have the same SCOP classes and high global structural similarity but different EC numbers (as different as at the top level of classification), PARSE was able to discriminate accurately by detecting local motifs. PARSE correctly predicted the EC number of 40 of 46 proteins, while correctly predicting up the third level in 3 proteins, up to the first level in one protein (*SI Appendix*, Table S1). We visually demonstrate an example with two proteins, UDP-glucose 4-epimerase and GDP-mannose 4,6-dehydratase, that share the same SCOP code. These two proteins have normalized TM-scores (52) of 0.83 and 0.89, but regardless, PARSE correctly distinguishes their EC numbers as 5.1.3.2 and 4.2.1.47 (*SI Appendix*, Fig S8).

### Precise Identification of Catalytic Residues.

**Fig. 3.**
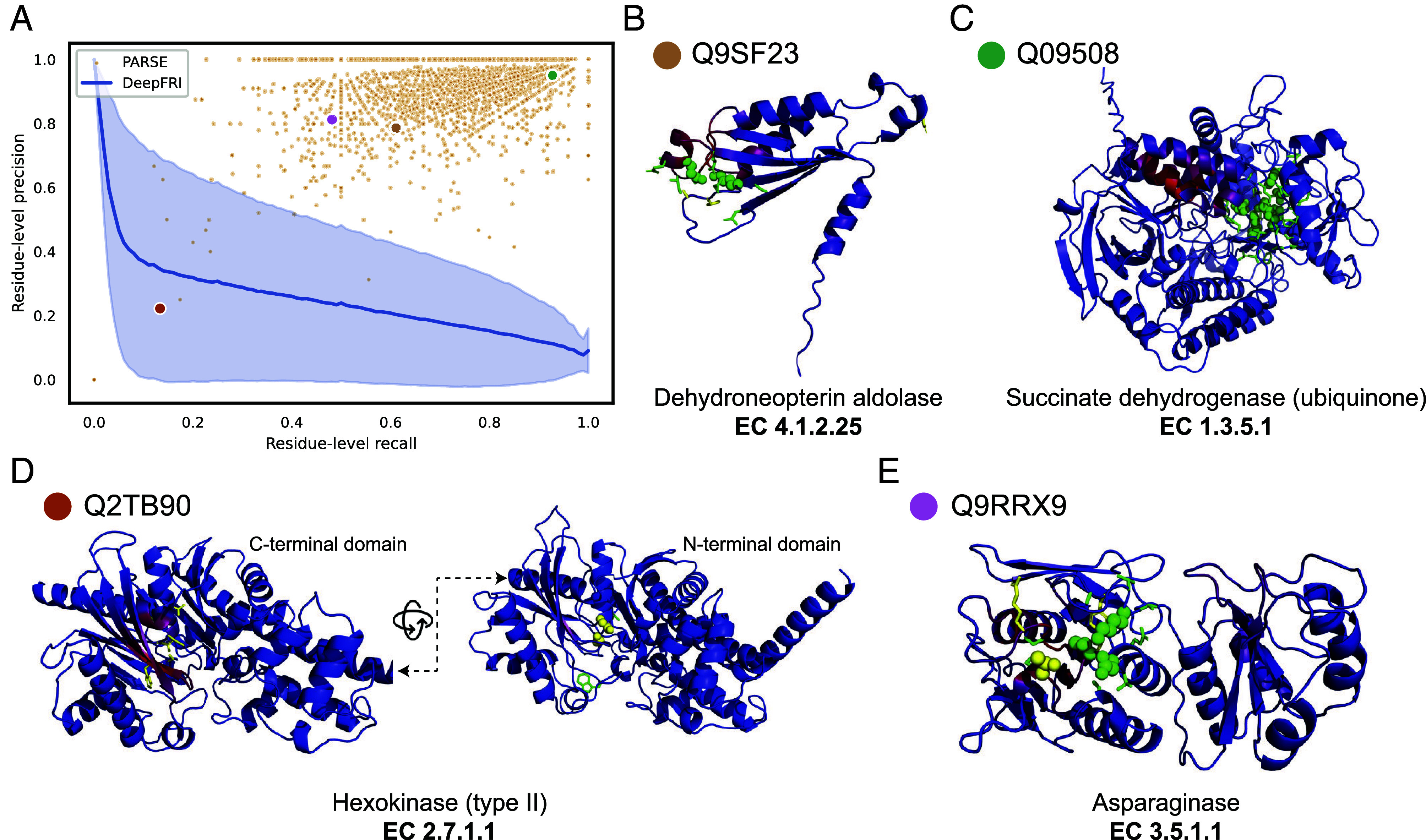
Annotation of enzyme active sites at amino acid resolution. (*A*) Residue-level precision and recall of active site identification over all correct predictions in the validation set. Each orange dot represents a single protein, and the four sampled proteins in (*B*–*E*) are labeled with colored dots. For comparison, DeepFRI performance is represented as a precision–recall curve, where the blue line is the average over all proteins and the shaded error bar is the SD. Four sampled structures, representing active site annotations by PARSE across proteins with diverse performance characteristics and enzymatic activities: (*B*) dihydroneopterin aldolase, (*C*) succinate dehydrogenase, (*D*) type-II hexokinase, and (*E*) asparaginase. In all examples, correctly identified active site residues are shown as green sticks. Correctly identified catalytic residues are shown as green spheres, and catalytic residues which are not identified by PARSE are shown as yellow spheres. Residues annotated by PARSE but not present in the reference site from CSA are shown as yellow sticks. The backbone cartoon is colored by DeepFRI’s gradient-weighted class activation map score, from blue (low) to red (high).

While accuracy in global function prediction is important for any method, we specifically designed PARSE to also identify the key functional residues involved in carrying out the protein’s function, a capability that is lacking in current methods. For enzymes, these key residues comprise the active site, which we define using the amino acids assigned a catalytic function by CSA and all immediate neighbors within 3.5 Å in the protein structure. To assess performance on active site residue annotation, we computed the residue-level precision and recall for each protein in the held-out test dataset that had a correct global function prediction (regardless of whether it was the top-ranked prediction). We compare only to DeepFRI because CLEAN does not produce residue-level predictions. Since DeepFRI produces a quantitative saliency score for each residue instead of a binary prediction, we compare performance for each protein across all possible score thresholds using precision–recall curves. Across the whole dataset, PARSE was able to identify active site residues much more accurately, with residue-level performance of most proteins exceeding that of DeepFRI regardless of threshold (Fig. 3*A*). Furthermore, PARSE achieves greater precision at equivalent recall in 584 of the 599 test proteins predicted correctly by both methods (*SI Appendix*, Fig. S2). In general, PARSE predictions are more specific than sensitive, with 58.7% of predictions achieving precision > 0.9 and recall > 0.5 but only 7.2% achieving both precision and recall exceeding 0.9 (4.0% and 0.0% of DeepFRI predictions reach these respective benchmarks at any threshold). However, this is partially due to our definition of active sites including both known catalytic residues and neighboring residues which may not be as functionally important. Indeed, we find that recall for detecting catalytic residues alone is significantly greater than recall over the entire active site, suggesting that the majority of residues missed by PARSE are noncatalytic (*SI Appendix*, Fig. S3).

To highlight the benefits of PARSE’s residue-level explainability for protein function prediction, we show four examples sampled from a range of performance characteristics and EC classes (Fig. 3*B*–*E*). Fig. 3*B* shows an example where we achieve only moderate precision and recall over the entire active site, but both the catalytic Lys and Glu residues are correctly identified. Some proteins, such as the succinate dehydrogenase shown in Fig. 3*C*, are annotated even more accurately—all eight catalytic residues are detected along with their closest neighbors. In both examples, the saliency predicted by DeepFRI is noticeably more diffuse and not centered around the catalytically active residues. Notably, in the latter case DeepFRI focuses instead on the binding site of the flavin adenine dinucleotide (FAD/FADH) cofactor, which is important mechanistically but not specific to this enzyme, being shared by all FAD-dependent flavoproteins.

In some cases, predictions which seem like misannotations may actually provide additional insight into the enzyme’s function and the limitations of existing databases. For example, Fig. 3*D* shows a hexokinase enzyme with two functional domains. Only the catalytic residues in the N-terminal domain were identified by CSA’s homology search, while both DeepFRI and PARSE correctly identify the equivalent residues in the C-terminal domain (representing CSA false negatives), resulting in reduced precision and recall. In another case, shown in Fig. 3*E*, PARSE misses the catalytic Thr16 residue in a putative asparaginase enzyme. However, this protein is also notably missing a key tyrosine (Tyr25 in reference PDB 3eca) that should interact with Thr16, suggesting that this protein may not in fact be catalytically active. The EC number was assigned to this protein in SwissProt based on sequence homology, which is generally insensitive to single-residue mutations, demonstrating the benefit of using local representations which capture the complex atomic environment around each residue.

### Scaling Annotation to the Full Human Proteome.

The AlphaFold Structure Database contains high-quality predicted structures for the proteomes of 48 organisms (34), offering an opportunity for structure-based functional annotation at scale. To this end, we applied PARSE to 21,575 proteins in the human proteome. Using the FDR cutoff of 0.001 tuned on the validation set, we produced 17,761 functional predictions for 8195 unique proteins. We observed that on this dataset, certain functions were predicted far more often than expected based on their known prevalence, even with the function-specific significance correction. Among these, almost half of the residues identified as functional had no overlap with the reference catalytic residues (*SI Appendix*, Fig. S4*A*). We find that these spurious predictions are driven largely by low-complexity structures in the AlphaFoldDB (see *SI Appendix*, Fig. S4*B* for examples), which are highly nonspecific and match many different reference structures. Because SwissProt is enriched for higher-complexity proteins, this type of spurious hit is not captured by our background distribution. Therefore, to increase the specificity of our proteome-wide predictions, we implement two filters: 1) at least 75% of reference catalytic residues must be identified by PARSE in the query structure, and 2) the all-atom RMSD between aligned catalytic residues of the two structures is less than five angstroms. The latter condition also requires that at least two catalytic residues must match the reference. These conditions reduced the number of predictions to 1,396, representing 1311 unique proteins.

**Fig. 4.**
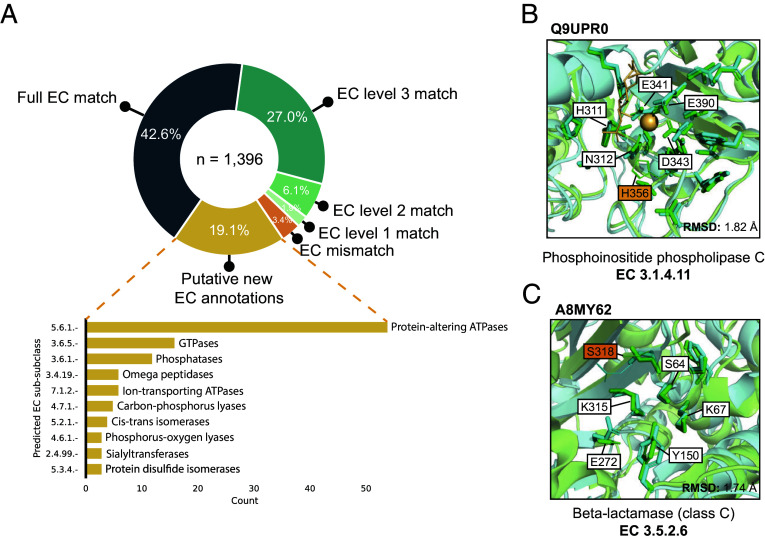
Expanding annotation coverage in the human proteome. (*A*) Comparison of PARSE predictions for AlphaFold structures in the human proteome to EC number annotations in UniProt, where available. Proteins are labeled as EC mismatch if the prediction does not match known annotations at any EC level, and putative new EC annotations are proteins with no EC numbers assigned in UniProt. For these hypotheses, we show the top 10 predicted enzyme classes at the third EC level. (*B*) Likely inactive PI-PLC with mutant catalytic residue H356T correctly not annotated by PARSE, and (*C*) putative class-C beta-lactamase predicted by PARSE. For both examples, reference structures from PDB are shown in green and query structures predicted by AlphaFold are shown in cyan. Residues identified as functional by PARSE are shown as sticks, and residues aligned to CSA residues but not annotated by PARSE are shown as lines. Catalytic residues are labeled using PDB numbering, and mismatches between query and reference are highlighted in orange. Proteins are aligned and RMSD is computed using catalytic residues only, including both backbone and side chain atoms.

Among these predictions, 69.6% matched an EC number assigned in UniProt to at least the third EC level (i.e. X.X.X.-), while a further 8.0% matched at either the first or second level (Fig. 4*A*). Only 47 predictions did not match any EC numbers; 12 of these resulted from an EC number transfer that produced a mismatch between UniProt and CSA annotations, while the remainder are either close homologs or bind similar ligands in the active site. The remaining 266 predictions correspond to proteins with no EC number in UniProt, representing putative new annotations. The majority of these are ATPases (notably myosins, kinesins, and chaperonins), G-protein GTPases, and phosphatases (notably HSP70 heat-shock proteins), reflecting the ubiquity of these protein families in cellular processing. Most of these are also well-annotated in UniProt but simply missing an EC number annotation, serving as positive controls and validating the ability of PARSE to identify missing annotations in sequence databases. All predictions for EC mismatch and putative new annotations are provided in Datasets S1 and S2.

We highlight two examples from the human proteome to showcase the utility of PARSE’s residue-level explainability and high functional specificity relative to existing methods. The first example, Q9UPR0, was annotated as a phosphoinositide phospholipase C (PI-PLC) due to high active site homology (Fig. 4*B*). However, it is likely inactive due to the substitution of a threonine residue for the catalytic histidine (His486 in Q9UPR0), an important feature which could not be detected by global methods. The second example, A8MY62, is much more sparsely annotated, with an assigned label of “putative beta-lactamase-like 1” (Fig. 4*C*). Beta-lactamases are a diverse class of enzymes with several subclasses (A, B, C, and D) which are further subdivided by catalytic mechanism and substrate specificity. All beta-lactamases share a single EC number (3.5.2.6), so they cannot be distinguished by existing methods for enzyme prediction which rely on EC number alone to define labels. PARSE, on the other hand, can predict any unique catalytic mechanism in CSA, allowing it to assign enzyme function with greater specificity. In this case, PARSE identifies A8MY62 as a class C beta-lactamase with no significant hits to other classes. Although a serine residue (Ser318 in the reference structure) is missing from the active site, mutagenesis studies have shown that mutations at this position do not affect the specificity of the enzyme (53, 54). The explainability of PARSE’s predictions thus facilitate confident assessments and provide biological intuition for computational functional predictions.

### Functional Hypotheses for Novel Folds in the Dark Proteome.

Protein research is strongly biased toward common and well-studied proteins, while the biological functions of thousands of others remain poorly understood (30). A recent clustering analysis of the entire AlphaFoldDB using Foldseek (55) identified over 40,000 clusters which could not be annotated using similarity to structures from known domain families. We hypothesized that PARSE’s ability to discover conserved local functional sites even with low fold-level similarity would make it ideally suited to discover novel enzymes in this dataset. Using the same filtering procedure as described for the human proteome to identify high-confidence hits, we annotated 34,015 representative structures from the dark proteome. This process predicted 183 putative novel enzymes from 51 different EC classes, including acylphosphatase (EC 3.6.1.7), isopenicillin-N synthase (1.21.3.1), nucleoside deoxyribosyltransferase (2.4.2.6), and ornithine cyclodeaminase (4.3.1.12). A full list of these predictions is provided in Dataset S3, and two examples are visualized in *SI Appendix*, Fig S5 *B* and *C*. We also provide predictions for the structures identified by Durairaj et al. (37) in Dataset S4, another recent work identifying structures in the dark proteome.

**Fig. 5.**
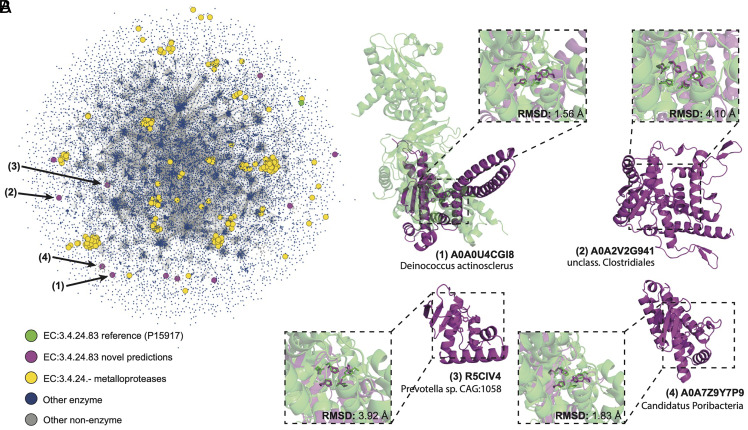
Annotation of dark proteome reveals novel metalloprotease folds. (*A*) Structural similarity of putative novel metalloproteases relative to the universe of known enzymes. New predictions are shown in purple, the CSA reference for EC number 3.4.24.83 in green, and other known metalloproteases in yellow. Blue dots are known enzymes in SwissProt, and edges are shown between proteins with similarity of less than 0.001 by Foldseek e-value. (*B*) Examples of four novel predictions (shown in purple), each aligned with the CSA reference structure (PDB 1PWV; green) using all atoms in the five catalytic residues (His686, Glu687, His690, Tyr728, and Glu735).

Interestingly, a large number of predictions belong to metalloprotease families (EC 3.4.24.-). In particular, 11 were predicted to belong to the EC number 2.4.24.83, a zinc-dependent endopeptidase which cleaves the N-terminus of mitogen-activated protein kinase kinases (MAPKKs). This enzyme and its homologs are key components of many bacterial toxins, making its inhibition attractive for therapeutic purposes (56). The predictions made by PARSE come from diverse bacterial species and exhibit unique structural folds, none of which show significant similarity to any known metalloprotease (Fig. 5*A*). In Fig. 5*B*, we show global and local active site structure for four of these predictions superimposed on the reference PDB structure based on the conformation of the five key catalytic residues. All predictions show high active site conservation despite the divergence in global fold, strongly suggesting a shared catalytic mechanism.

## Discussion

To improve widespread acceptance and trust in artificial intelligence in biology, it is important for methods to provide not only accurate predictions but also explanations that correspond to biological intuition. In this work, we propose an approach to protein function annotation that combines the advantages of pretrained protein representations with prior biological knowledge and statistical methods to improve explainability while retaining high predictive performance. In contrast to standard supervised learning approaches which start with global classification and then attempt to explain these predictions post hoc, PARSE is a bottom–up approach that starts by identifying putative functional sites at the residue level before aggregating predictions over the entire protein. This formulation is explainable by construction, since any global prediction can be traced back to each contributing residue, and provides a meaningful improvement over the post hoc Grad-CAM explainability method used in DeepFRI and other similar methods. This approach is also stronger than methods which rely on single residue-level comparisons (43) because it combines signal over multiple sites which may have individually moderate similarity. In general, we believe that it is important to explore alternative approaches to making AI-enabled predictions that are more mechanistically justified and human-comprehensible, and PARSE represents a meaningful step in this direction.

The use of local, site-level similarities rather than protein-level similarities has several benefits for functional discovery in addition to providing explainable predictions. First, it enables identification of conserved functional motifs even when global sequence and structure are highly divergent, as in the case of the dark proteome metalloproteases shown in Fig. 5. Second, it is possible to predict function even if only part of the protein’s structure is known with high confidence. In many cases, AlphaFold2 produces predictions with large, fragmented regions of low-confidence loops interspersed with high-confidence globular domains. By only matching on local high-confidence regions, we can avoid the noise introduced by inaccurate predictions in other regions of the structure.

A major strength of the PARSE algorithm is its modularity and flexibility; each component can be easily adapted based on the biological task. For example, a new reference database could be constructed for or any problem where residue-level knowledge bases exist (e.g. ligand-binding sites, posttranslational modifications), or new functions could be added manually based on new experimental data. Adjusting the significance threshold controls precision and recall depending on which is more important based on the task at hand. The GSEA-like scoring function could be replaced with any statistical method which returns enrichment scores for each class and the key residues which contribute to the prediction. Improvements here may help to increase statistical power and reduce the influence of low-complexity structures which cause false positives in our proteome-wide scans. This is a known weakness of the K-S test when used in a preranked setting, which tends to overestimate significance for sets with high internal correlation between elements (57). Our function-specific empirical significance calculation largely addresses this problem but may still produce false positives for proteins that are outside the background distribution (e.g. AlphaFold predictions for the dark proteome), particularly with very simple helical secondary structures.

Finally, the local representation could also be adapted for other data types; while COLLAPSE is the primary embedding method for local protein sites, any pretrained local representation could be used instead. Indeed, the recent success of ESMFold (22) has demonstrated that large protein language models (PLMs) implicitly learn local representations that enable atomic-level prediction protein structure. To test whether the underlying residue-level embeddings could enable functional annotation under the PARSE framework, we implemented PARSE with embeddings from ESM2 and find that the performance is comparable to that of COLLAPSE embeddings (albeit slightly slower due to larger embedding sizes) (*SI Appendix*, Fig. S6). This demonstrates the generalizability of PARSE across representation types and suggests that as protein representations improve, so will the ability of PARSE to detect remote functional relationships. We note that while PLM embeddings are computed only on sequence, the 3D structure is still important for PARSE-it is still required to define the residues in the active site, and is critical to the explainability of the method, since it is important to examine the residue-level predictions in their structural context to understand the predictions and build biological intuition.

The most significant limitation of PARSE is its reliance on a high-quality database of residue-level labeled data. The Catalytic Site Atlas is an excellent resource for this purpose, but it is limited to 940 enzymes and has not been updated for several years. This highlights the importance of expanding site-level as well as global protein annotation databases as biological knowledge increases. Importantly, since PARSE requires only one reference example to make predictions, it is relatively straightforward to curate larger datasets without requiring high-throughput experiments. As methods for extracting and synthesizing knowledge from across biomedical literature improve, we anticipate that large-scale databases will become more widespread, expanding the coverage of site-based methods such as PARSE for new function discovery.

For enzyme function prediction, PARSE recapitulates known SwissProt annotations much more accurately than DeepFRI, the best-performing existing method which provides residue-level explanations. The improvement is especially notable for rare and understudied enzyme classes, an important characteristic which can be attributed to the one-shot nature of PARSE’s database comparisons. The best-in-class global method, CLEAN, also has few-shot ability due to its contrastive learning objective. Although it performs better than PARSE on our rare enzyme dataset, it is important to note that the publicly available implementation of CLEAN was pretrained on a 100% nonredundant clustering of SwissProt, so even the rare enzymes are in-distribution for this model.

At the amino acid level, we perform the largest-scale quantitative evaluation to date of residue-level performance for machine learning based protein function prediction models. We find that residue-level annotations provided by PARSE correspond much more accurately to the catalytic site of the enzymes than DeepFRI’s class activation mapping approach. This is in agreement with previous studies which note the pitfalls of post hoc gradient-based explainability methods (46, 47). Gradient-based methods are also becoming increasingly unsuitable as large-scale foundation models (58) become increasingly widespread in biology, since such models are run almost exclusively in inference mode and often do not provide access to internal model weights. We anticipate that methods such as PARSE, which combine pretrained embeddings with prior biological knowledge and interpretable statistics, will be critical for making explainable and trustworthy predictions in this paradigm.

The release of AlphaFoldDB provides an unprecedented opportunity to apply structure-based predictors to discover new biological functions at proteome scale. On this largely unexplored dataset, especially the entirely novel folds in the dark proteome, the residue-level explainability of PARSE is especially important for evaluating predictions, as we show through several illustrative examples. Most notably, we find strong evidence for several new bacterial metalloproteases which have highly divergent structures and sequences but retain a strongly conserved active site. These findings illustrate the potential of local representations combined with large structural databases to gain functional insights, which may help our understanding of pathogenic processes and aid in the development of more potent and specific therapeutics. As protein structure predictors improve and databases continue to expand to hundreds of millions of metagenomic proteins (22), we expect that methods such as PARSE will become even more powerful tools for biological discovery.

## Materials and Methods

### Reference Database Construction.

Our reference database consists of the manually curated residue-level annotations for enzymes in the Catalytic Site Atlas. We extract the relevant chain and catalytic residue identifiers from the reference PDB entry for each enzyme class. Since the average number of catalytic residues for each structure is less than five, which is not enough on its own to achieve good statistical power in large-scale searches, we expand the enzyme active site to include all residues which have at least one atom within 3.5 Å of any atom in a catalytic residue. This threshold was chosen to capture any residue that may interact with a catalytic residue (e.g. via hydrogen bonding). We remove all ligands, waters, and other heteroatoms from the reference chain. Then, we embed the structural microenvironment surrounding each active site residue to a 512-dimensional numeric vector using COLLAPSE, which considers all atoms within a 10 Å radius of the predefined functional center of each amino acid (43). The result of this process is a database consisting of 26,157 residues corresponding to 939 unique functional sites.

### Evaluation Dataset Creation and Processing.

We evaluated on AlphaFold predicted structures for known enzymes in SwissProt, starting with the sequence homologs provided by CSA, which are identified by searching each reference sequence against UniProt using PHMMER (59) with an e-value cutoff of $1\times 10^{-6}$. Conserved catalytic residues in these alignments are then annotated to serve as a ground truth for residue-level predictions. Since these results are based on sequence similarity, there are many false positives (e.g. proteins from related families with different catalytic mechanisms). Therefore, to create a “gold-standard” dataset for evaluation, we included only proteins with a curated SwissProt EC number that perfectly matches the EC number for the reference CSA entry. We then redundancy-reduced this dataset using 50% sequence identity clusters from Uniref50 (60) to ensure that each protein belongs to a different sequence cluster. We also removed all proteins which share a sequence cluster with any protein in the reference database. This process resulted in 17,262 unique proteins representing 17,779 total function annotations. To create a held-out test set which would not be used for tuning the significance threshold, we binned proteins by the frequency of their corresponding enzyme classes in SwissProt. All enzymes with at least 100 examples were used for validation (269 unique functions) and the remainder were reserved for testing (425 unique functions). For all datasets derived from AlphaFoldDB (SwissProt validation and test, human proteome, and dark proteome), the environment around every residue with high or very high confidence (pLDDT ≥ 70) was embedded using COLLAPSE and stored along with corresponding metadata (e.g. UniProt ID, residue IDs, pLDDT). Links to download these precomputed datasets are provided along with the code in our GitHub repository.

### PARSE Implementation Details.

The PARSE algorithm consists of three main steps, outlined here in detail and shown in Fig. 1.

1. Embed input protein. Every residue of the input structure is embedded using COLLAPSE (43), using the same parameters as in the construction of the reference database. If the input structure is an AlphaFold predicted structure, we only consider residues with pLDDT ≥ 70 to reduce the influence of low-confidence structural regions.
2. Rank reference residues by similarity to input protein. First, we compute the pairwise cosine similarity between the database embeddings and the input protein embeddings. Then, for each database site we identify the maximum similarity to any residue in the query. Database sites are then sorted by this maximum similarity to produce the final ranked list. This process also produces the mapping between database sites and the nearest residue in the query which is used to compute final residue-level annotations.
3. Identify enriched classes and key functional residues. We compute an enrichment score (ES) statistic for each function class F by walking down the ranked list and increasing or decreasing a running sum statistic S depending on whether the database residue is in F or not in F, respectively. We use the same increment and decrement formulas as in GSEA (49) to compute S, and the ES is similarly calculated as a weighted Kolmogorov–Smirnov statistic using the maximum deviation of S from zero. The raw ES should not be used to directly rank functional classes due to the differences in the null distribution of scores within each class, necessitating the calculation of class-specific significance scores (57). In standard preranked GSEA, statistical significance is assessed by permuting the gene labels; however, this is known to overreport significance when there is high correlation within gene sets. We observe the same phenomenon for our dataset, so we instead estimate significance using a function-specific empirical ES distribution. Specifically, for each function class we measure the ES over all proteins in our validation and test datasets that are not annotated with that function in SwissProt. The empirical *P*-value for a new enrichment score s is then computed as $p=\frac{\sum_{i}^{\left|D\right|}\left(s>d_{i}\right)}{\left|D\right|}$, where $d_{i}\in D$ are the individual ES over the background distribution D. This approach is similar in spirit to the permutation of association scores proposed for multisample GSEA by Tian et al. (61) and significantly improves the sensitivity and specificity of the resulting predictions. To correct for multiple hypothesis tests, we control false discovery rate (FDR) using the Benjamini–Hochberg procedure.

### Baseline Methods.

Our goal was to compare PARSE to existing methods out-of-the-box, as they would be used by practitioners. Therefore, we used the inference scripts and pretrained model weights provided in the GitHub repositories for DeepFRI (https://github.com/flatironinstitute/DeepFRI) and CLEAN (https://github.com/tttianhao/CLEAN) directly. For DeepFRI, we preprocessed all PDB files to produce distance maps and sequence embeddings and predicted EC numbers using the default model architecture: three MultiGraphConv convolutional layers with dimension 512, followed by a linear encoder of dimension 1,024. Final predictions are assessed using the default predicted probability cutoff of 0.1. For CLEAN, we preprocess the dataset into fasta files by unique chain, use the default *split100* pretrained model weights, and make predictions using the maximum separation procedure. Note that the set of possible EC number labels DeepFRI is trained on are not identical to those used for CLEAN; this is reflected in the baseline comparison results, particularly where less specific labels are preferred by DeepFRI (i.e. third level EC number). For BLASTp comparisons, we search each validation and test protein against the reference database using default settings and an e-value cutoff of 0.01. Since PARSE uses enzyme class definitions defined by catalytic mechanism in CSA-which is more specific than EC numbers in some cases-for all baseline comparisons, we convert the CSA class predicted by PARSE to its corresponding EC number.

### Human and Dark Proteome Datasets.

The human proteome dataset was downloaded from AlphaFoldDB (https://www.alphafold.ebi.ac.uk/download) on July 20, 2021. The UniProt annotations for proteins in the dark proteome were derived from the data provided by Barrio-Hernandez et al. (36). We used the reference structures for each dark cluster with average pLDDT > 90 downloaded from the AlphaFoldDB website on October 21, 2022. All predicted structures for both human (n=21,575) and dark (n=34,015) proteomes were processed as described for the SwissProt evaluation dataset, removing structures that had no high-confidence residues and embedding using COLLAPSE. We also included four baselines that make less sophisticated and more direct use of the COLLAPSE embeddings and their similarities compared to PARSE. The baselines are as follows: 1) “Max similarity:” We picked the maximum similarity between any two residues (one in the reference database and the other in the query) and assigned the functional label of the reference database residue with the highest similarity score. 2) “Top k% mean similarity:” We carried out the same ranking of all the reference database residues based on their maximum similarity and then computing the mean similarity of the top k% most similar residues in each functional class. We assigned the label of the functional class whose average top k% similarity is the highest. We repeated this for six different k values, ranging from 10 to 40. 3) “Direct MWU:” We used the same ranking of the database residues and binarized the ranked residues as “in-class” or “out-of-class.” Upon binarization, we conducted a one-sided Mann–Whitney *U* test for each of these classes to test for a statistical difference in the ranks, with the alternative hypothesis being that residues belonging to the functional class are preferentially ranked nearer the top of the similarity list (i.e. their similarity scores are larger) than residues outside the class. We picked the functional class with the lowest *P* value, indicating the most significantly ranked near the top. 4) “MWU with background:” We replicated the PARSE workflow, only replacing the Kolmogorov–Smirnov statistic with Mann–Whitney *U*.

### Active Site Alignment to Reference Structures.

We use structural conservation of the active site as one piece of evidence to support a functional prediction and filter down proteome-scale results. We quantitatively evaluate conservation using RMSD between all matching catalytic residues in the active site. To compute this, we identify PARSE annotations with an exact amino acid match to the corresponding catalytic residue in CSA and extract the 3D coordinates of all atoms (including side chain and backbone) in these residues from both reference and query structures. We then align these sets of coordinates using the Kabsch algorithm (62) and compute RMSD between all atoms.

## Supplementary Material

Appendix 01 (PDF)

Dataset S01 (CSV)

Dataset S02 (CSV)

Dataset S03 (CSV)

Dataset S04 (CSV)

## Data Availability

Embedding data have been deposited in Zenodo: Data for PARSE (https://doi.org/10.5281/zenodo.8437086). All other data are included in the manuscript and/or supporting information. Previously published data were used for this work (1–3, 5–7, 32, 34, 36, 37).
